# Effect of Shrub Components on Soil Water and Its Response to Precipitation at Different Time Scales in the Loess Plateau

**DOI:** 10.3390/ijerph20064722

**Published:** 2023-03-07

**Authors:** Jianbo Liu, Guangyao Gao, Bing Zhang

**Affiliations:** 1 Tianjin Key Laboratory of Water Resources and Environment, Tianjin Normal University, Tianjin 300387, China; 2 State Key Laboratory of Urban and Regional Ecology, Research Center for Eco-Environmental Sciences, Chinese Academy of Sciences, Beijing 100085, China; 3 National Observation and Research Station of Earth Critical Zone on the Loess Plateau in Shaanxi, Xi’an 710061, China

**Keywords:** plant components, rainfall, removing canopy, soil water storage, temporal dynamic, vegetation restoration

## Abstract

Water shortages have become the major limiting factor for ecological protection and sustainable development in the Loess Plateau. Few studies have focused on the effects of different plant components on soil water and its response to precipitation at different time scales. This study conducted an observation of shrub plants with three treatments (natural condition (NC), canopy + roots after removing the litter (CR), and only roots (OR)) to monitor the dynamics of soil water during the rainy season of an extreme drought year in 2015. The results showed that the soil moisture content (SMC) and soil water storage (*W*) had a trend of OR > CR > NC. The response of the SMC to precipitation was gradually decreased and delayed for longer with increasing soil depth. Daily precipitation >10 mm was the threshold to trigger an SMC response below 20 cm of depth. The thresholds of precipitation to increase *W* were 2.09–2.54 mm at the daily scale and 29.40–32.56 mm at the monthly scale. The effect of precipitation on *W* and its change (∆*W*) also depended on the time scales. At the daily scale, precipitation only explained 1.6%, 0.9%, and 2.4% of the *W* variation in NC, CR, and OR, respectively. However, precipitation was more important for ∆*W*, making a contribution of 57.6%, 46.2%, and 56.6%, respectively, and the positive ∆*W* induced by precipitation happened more easily and frequently at deeper depths in OR. At the monthly scale, the contribution of precipitation to ∆*W* increased to 75.0%, 85.0%, and 86%, respectively. The ∆*W* of the whole rainy season was OR > NC > CR. Precipitation of the monthly scale displayed higher contributions to soil water than that of the daily scale. Plant components had different influences on soil water and its response to precipitation, which was strengthened by the roots, weakened by the canopy, and neutralized by the litter. Regular cutting of the canopy at the single-shrub scale may help increase water storage, which is useful for vegetation management and hydrologic regulation.

## 1. Introduction

Soil water is a key bridge connecting the upward and downward movement of the water cycle, which plays an important role throughout the soil–plant–atmosphere continuum [1]. Soil water has become the major limiting factor for ecological protection and sustainable development in the Loess Plateau, and its importance is becoming more and more remarkable due to the huge depletion of soil water after the successful application of the Grain-for-Green Project in 1999 [2]. Large-scale vegetation restoration has controlled soil erosion well [3,4,5], but it has consumed a large amount of soil water, which has changed the distribution of soil water and has even led to the formation of a dry soil layer [6,7]. This water-shortage phenomenon has gradually aggravated the ecosystem’s vulnerability and constrained the ecological sustainability of the Loess Plateau [2,7]. Therefore, it is essential to understand the changing mechanism of soil water and its influencing factors.

Precipitation is a unique source of soil water replenishment and has significant effects on soil water processes, including the intensity, amount, duration, pattern, etc. [8,9,10,11,12,13]. Dai and Wang [14] found that <7.0 mm rainfall events had no effect on improving soil water in the typical steppe of northern China, as it was consumed by evaporation, surface vegetation interception, and other losses. Meanwhile, with an increase in soil depth, the needed effective amount of rainfall events gradually increases and the response of soil water after rainfall events is gradually delayed for a longer time. Ge et al. [10] also found that only rainfall events larger than 5 mm could affect the 10 cm deep soil water in the Loess Plateau. They also noted the importance of rainfall patterns and observed that higher intensity and smaller amount of rainfall showed a quick surface soil water response, while a larger amount of rainfall had a deeper and faster influence. The threshold phenomenon, hysteresis effect, and rainfall pattern effect of rainfall events on soil water were also verified by Zhao et al. in the Loess Plateau [12]. They demonstrated that the response depth of soil water was only 40 cm for most events and that only a few extreme events could affect deeper soil. However, the depth and time of soil water response may differ from region to region. For example, a heavy event of more than 160 mm triggered a 0–160 cm response in one day on Beijing Mountain [15]. Previous studies have focused more on rainfall events, and not enough attention has been paid to the daily and monthly scales.

Plant components have different and important functions in hydrological processes. The aboveground canopy redistributes rainfall into interception, throughfall, and stemflow; first, by changing the dropping process of rainfall; second, by reducing net radiation to the soil surface via its shadow; and third, by decreasing the soil water due to an increase in canopy transpiration [16,17,18]. The surface litter intercepts rainfall and runoff as well, delaying runoff generation and preventing rainfall and runoff from directly reaching the soil but also storing water to maintain a longer period of infiltration after the rainfall and runoff processes [11,19,20]. The underground roots show a significant influence on water infiltration, where root channels can largely improve the infiltration capacity, and the improvement of soil properties by roots also enhances water storage, both of which lead to greater and easier soil water infiltration [19,21,22]. However, previous studies have paid more attention to the effect of different vegetation types [10,12,23], and there are few studies on the role of different plant components and their effects on soil water’s response to precipitation at different time scales.

The Loess Plateau is a critical zone that faces huge human–water–ecology relationship challenges, so soil water conservation is an urgent practical demand and a scientific problem that requires more attention. In order to clarify the dynamics of soil water and its response to precipitation after vegetation restoration, we monitored the soil moisture content (SMC) of a shrub species in a typical small catchment in the Loess hilly and gully region with different treatments of plant components. The objective of this paper is to analyze the temporal dynamics of SMC at different depths, to reveal the response of soil water storage (*W*) and its change (∆*W*) to precipitation at different time scales, and to understand the effect of plant components on the above processes.

## 2. Materials and Methods

### 2.1. Study Area

**Figure 1:**
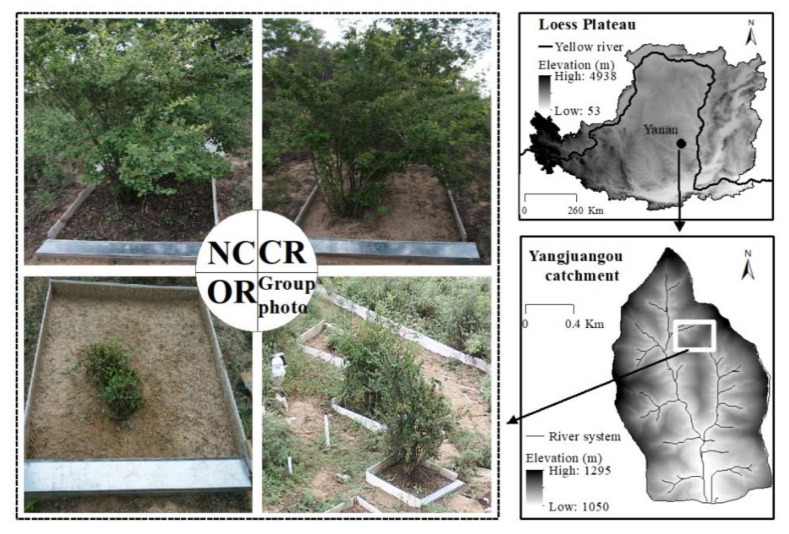
Location of the study area and the plots with different treatments. NC shows the natural condition including the canopy, litter, and roots of plants. CR indicates the state of the canopy + roots after removing the litter. OR represents only the roots after removing both the canopy and litter.

The study area is located in the Yangjuangou catchment (36°42′ N, 109°31′ E), which is a typical hilly and gully catchment in the central region of the Loess Plateau, China (Figure 1). The catchment has a total area of 2.02 km^2^ and an elevation that ranges from 1050 to 1298 m. It is a typical semi-arid area with an average annual precipitation of 536 mm between 1951 and 2016, ranging from 330 to 959 mm. The average annual T is 9.4 °C [24]. Precipitation is mainly concentrated between June and September, accounting for more than 70% of the total annual precipitation, with large inter-annual variations. The soil is a *Calcaric Cambisol* with a maximum depth of approximately 200 m. It has a uniform texture and is easily erodible. The dominant vegetation in the catchment consists of replanted vegetation due to the Grain-for-Green project launched in 1999, including forest, shrub, and grass types. The dominant species of different vegetation types contain *Robinia pseudoacacia*, *Prunus armeniaca*, *Spiraea pubescens*, *Hippophae rhamnoid*, *Stipa bungeana*, *Artemisia sacrorum*, *Andropogon* L., *Artemisia scoparia*, and so on. *Spiraea pubescens* is a typical shrub species that is widely distributed in the Loess Plateau and has a high canopy coverage of more than 80%, a high litter coverage of almost 90%, a canopy height of 1–2 m, and a size of about 1.0 m.

### 2.2. Field Experiments

A typical *Spiraea pubescens* shrub was selected to measure soil moisture under different treatments and depths. Three types of treatments were conducted at the single-plant scale (Figure 1), including (1) the natural condition (NC) without any artificial disturbance; (2) the canopy + roots (CR) after removing the litter; and (3) only the roots (OR) after removing the canopy and litter. Each treatment had three repeats. Microplots were established for each shrub. The plots had similar slope gradients of approximately 22–24° and a similar slope aspect of northeast. To avoid the effects of surrounding surface runoff and subsurface flow, PVC and plastic material sheets (at a depth of 50 cm) were used as the boundaries of plots. The size was 1.0 m × 1.0 m, which is consistent with the size of a single shrub.

Three microplots with different treatments at the same slope (Figure 1, group photo) were selected to install EC-5 sensors to measure the volumetric soil moisture content (SMC, m^3^/m^3^) at 5, 10, 20, and 40 cm depths. The data were sampled at 30 s intervals and the averaged value was recorded at 5 min intervals by the data logger (U30, Onset Computer Corp., Bourne, MA, USA). Precipitation (P, mm) was measured by a tipping bucket rain gauge (RG3-M, Onset Computer Corp., Bourne, MA, USA) with a resolution of 0.2 mm. The rain gauge was placed in an open area near the microplots. The observation period was from 9 June to 19 September of 2015 for SMC and precipitation. The rain gauge was blocked by litter, dust, and other things on 11 September, so manual measurements were used to replace the automatic measurements at the daily scale.

### 2.3. Data Analysis

The SMC per day was the average value of SMC monitored at 5 min intervals during each day.

The mean soil moisture content (*SMC_m_*, m^3^/m^3^) was the weighted average value of *SMC* at different depths:

(1) $${\mathrm{SMC}}_{m}=\frac{\sum^{\;}{\mathrm{SMC}}_{i}{\;\times \;h}_{i}}{h}$$

where *SMC_m_* is the mean soil moisture content for each treatment at 0–40 cm depth (m^3^/m^3^), *SMC_i_* is the soil moisture content at depth *i* (m^3^/m^3^), *h_i_* is the thickness at depth *i* (cm) (*h_i_* = 5, 5, 10, and 20 cm, respectively), and *h* is the total depth of 40 cm.

The soil water storage (*W*, mm) was calculated as:

(2) $$W_{i}{=\mathrm{SMC}}_{i}{\;\times \;h}_{i}\;\times \;10$$

(3) $$W=\sum W_{i}$$

where *W_i_* is the soil water storage at depth *i* (mm) and *W* is the total soil water storage at 0–40 cm depth.

The change in soil water storage (∆*W*, mm) was controlled by soil water processes, including precipitation infiltration, evapotranspiration loss, and infiltration into deeper soil layers. However, soil water always remained stable below 40 cm depth in this area during one rainy season and did not respond to precipitation infiltration according to the results of a previous study [23] and the analysis of the results presented in part 3.2 of this paper. Thus, we assumed that the soil water at 0–40 cm depth did not have exchanges with deeper soil layers. The ∆*W* in this paper was only controlled by the input of precipitation infiltration and the output of evapotranspiration, which was the difference between the initial *W* and the final *W* at different time scales. The daily ∆*W* was the difference in *W* each day, the monthly ∆*W* was the difference in *W* over the course of a month from the initial day to the final day, and the total ∆*W* of the whole rainy season was the difference in *W* between the initial week and the final week.

One-way ANOVA and the LSD test (*p* < 0.05) were used to analyze the differences of *SMC*, *W*, and ∆*W* between different treatments or months. When both the homogeneity test of variance and normal transformation of initial data failed, the Brown–Forsythe test and Dunnett’s T3 test (*p* < 0.05) were chosen to compare the differences between treatments. Spearman’s correlation analysis was used to analyze the correlation between precipitation and soil water at different depths. A linear fit between the precipitation and ∆W at different time scales was conducted to calculate the determination coefficient (R^2^), which explained the contribution of precipitation to Δ*W* variation. All statistical analyses were performed using SPSS 19.0 software.

## 3. Results

### 3.1. Dynamics of the Mean Soil Moisture (SMC_m_) at the Daily Scale

**Figure 2:**
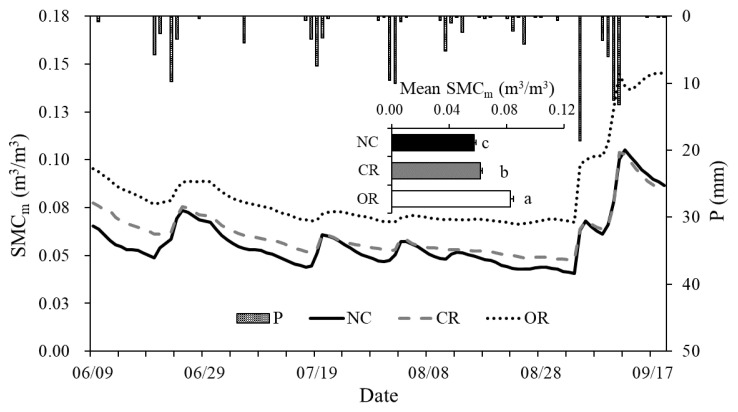
Dynamics of the SMC_m_ for different treatments and precipitation during the rainy season in 2015. Different letters show a significant difference at *p* < 0.05 between natural condition (NC), canopy + roots (CR), and only roots (OR).

The temporal dynamics of the *SMC_m_* of the three treatments and precipitation at the daily scale are shown in Figure 2. There were significant differences in the *SMC_m_* between different treatments (i.e., NC, CR, and OR). The OR treatment had the highest *SMC_m_* throughout the whole rainy season (*p* < 0.05), with an average value of 0.082 m^3^/m^3^, ranging from 0.067 to 0.145 m^3^/m^3^. The NC treatment showed the lowest *SMC_m_* before 9 September and was then slightly higher than the CR treatment until 19 September, with an average value of 0.057 m^3^/m^3^, ranging from 0.041 to 0.105 m^3^/m^3^. The CR had an intermediate average *SMC_m_* of 0.062 m^3^/m^3^, ranging from 0.048 to 0.104 m^3^/m^3^. In addition, the one-way ANOVA analysis showed that the *SMC_m_* of the three treatments had significant differences, with the order being OR > CR > NC (*p* < 0.05).

The temporal responses of the *SMC_m_* to precipitation in the three treatments were similar and positive. In particular, the greater the daily precipitation, the timelier and more sensitive the daily *SMC_m_*. Following rainfall, the *SMC_m_* gradually decreased over time, where the fastest and steepest decline was found in NC, followed by CR and then OR before 9 September. The OR treatment showed a different trend in the *SMC_m_* dynamic at the end of the observation period.

### 3.2. Dynamics of Daily SMC at Different Depths

**Figure 3:**
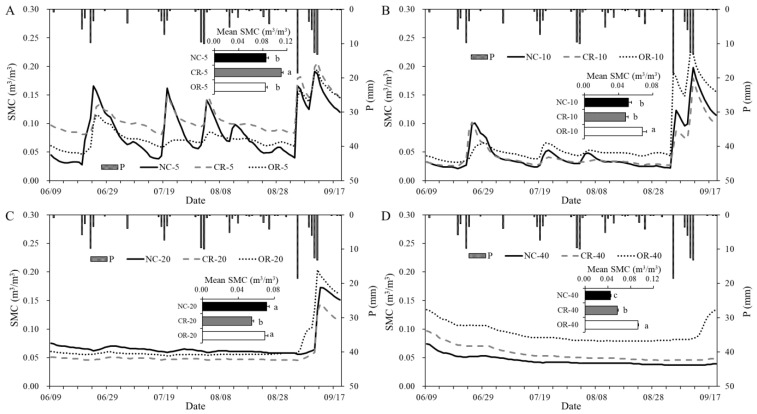
Dynamics of the daily SMC and its mean value at different depths for different treatments. (**A**–**D**) describe depths of 5 cm, 10 cm, 20 cm, and 40 cm, respectively. Different letters show a significant difference at *p* < 0.05. P: precipitation, NC: natural condition, CR: canopy + roots, OR: only roots.

The temporal dynamics of the daily SMC at different depths is shown in Figure 3, which shows different trends for the three treatments. At 5 cm depth (Figure 3A), the CR showed significant differences from NC and OR (*p* < 0.05), with the highest average SMC being 0.111 m^3^/m^3^, and the SMC ranged from 0.081 to 0.207 m^3^/m^3^. The average values of NC and OR were similar (0.086 and 0.084 m^3^/m^3^, respectively) and unsignificant. At 10 cm depth (Figure 3B), the CR had the lowest SMC of 0.048 m^3^/m^3^, which was not significantly different from NC (0.051 m^3^/m^3^). The average value of OR (0.068 m^3^/m^3^) was significantly higher than that of the others (*p* < 0.05), ranging from 0.032 to 0.225 m^3^/m^3^. At 20 cm depth (Figure 3C), the CR also showed the lowest average SMC of 0.055 m^3^/m^3^, differing significantly from the others (*p* < 0.05) and ranging from 0.045 to 0.144 m^3^/m^3^. At 40 cm depth (Figure 3D), the differences in the SMCs of the different treatments were significant, with a trend of OR > CR > NC.

The results also indicate that the SMC at different depths had different responses to daily precipitation. SMC at 5 cm depth showed the highest sensitivity to precipitation with no delay time, and the NC exhibited the largest amplitude of variation and the deepest decreasing curve after rainfall over time. At 10 cm depth, the curves of SMC were more stable, and the moisture peaks occurred later with 0–2 day delay before September. At 20 cm depth, the fluctuation of SMC was lower, and the weak peaks were delayed by about 5 days more before September, but the responses to precipitation became huge in September, especially for the OR treatment. At 40 cm depth, SMC showed stable curves before September, and only OR had an obvious increase after being delayed by 8 days in September. Briefly, the intensity and timeliness of the SMC response to precipitation showed a trend of 5 > 10 > 20 > 40 cm, but this was influenced by different treatments and the precipitation amount.

### 3.3. The Relationships between W, ∆W, and Precipitation at the Daily Scale

The correlation between soil water storage (*W*) and its change (∆*W*) with precipitation from June to September at daily scale is shown in Table 1. For the total depths of soil, the *W* of every day had insignificant and negative correlations with precipitation, and the absolute values of the correlation coefficients gradually decreased from OR (0.043) to CR (0.125) to NC (0.167). On the contrary, the ∆*W* of every day showed a significant and positive correlation with precipitation (*p* < 0.01), and the coefficients gradually increased from OR (0.384) to CR (0.419) to NC (0.504).

For different soil depths, precipitation still had no significant correlation with daily *W* at 0–10 cm depth, but it became more relevant to daily *W* below 10 cm depth, which partially reached the significant level (*p* < 0.05). Moreover, the correlations between daily precipitation and ∆*W* at 5, 10, and 40 cm depths were both positive and significant (0.238–0.477) (*p* < 0.05) but were negative and insignificant at 20 cm depth.

**Figure 4:**
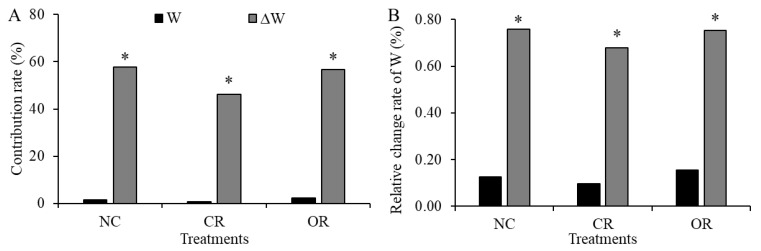
The contribution rate of precipitation to the *W* and ∆*W* variation (**A**) and their relative change rates (**B**) at the daily scale. * indicates the significance at the *p* < 0.05 level. *W*: soil water storage, ∆*W*: the change in soil water storage, NC: natural condition, CR: canopy + roots, OR: only roots.

In Figure 4A, the determination coefficient (R^2^) of regressions was used to describe the contribution rate of precipitation to the variation of *W* and ∆*W* at the daily scale. Precipitation only accounted for 1.6%, 0.9%, and 2.4% of the contribution rate to the variation of *W* in NC, CR, and OR, respectively. However, the contribution rate of precipitation to the variation of ∆*W* significantly increased to 57.6%, 46.2%, and 56.6%, respectively. Standardized regression coefficients were used to represent the relative change rate of daily *W* and ∆*W* when precipitation changed by 1% (Figure 4B). The relative change rates of *W* in NC, CR, and OR were 0.13%, 0.10%, and 0.16%, respectively, and they were 0.76%, 0.68%, and 0.75% for ∆*W*, respectively. This indicates that the ∆*W* was more strongly controlled and easily influenced by precipitation, and the CR treatment showed the lowest response of ∆*W* to precipitation at the daily scale.

### 3.4. Responses of ∆W to Precipitation on Rainy Days

**Figure 5:**
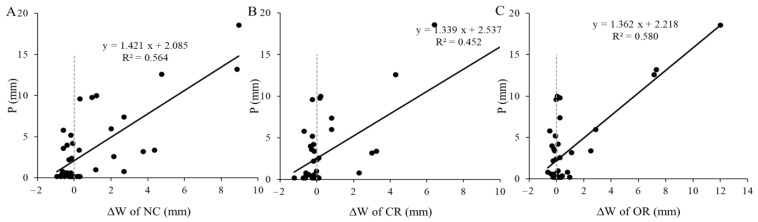
Relationships between ∆*W* and precipitation on rainy days in the NC treatment (**A**), CR treatment (**B**) and OR treatment (**C**). The dashed line is the zero-value dividing line. P: precipitation, ∆*W*: the change in soil water storage, NC: natural condition, CR: canopy + roots, OR: only roots.

**Figure 6:**
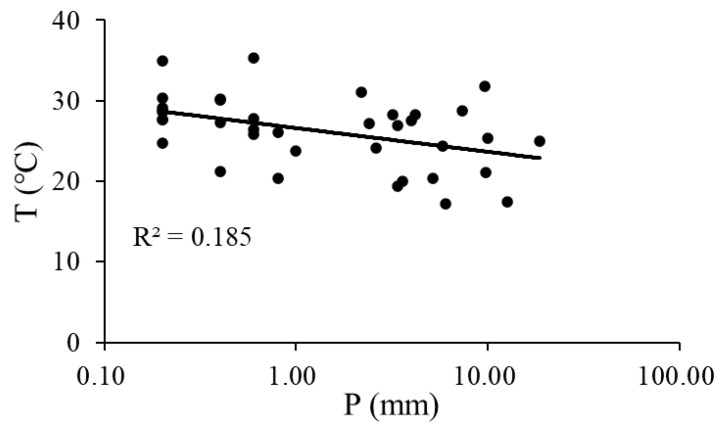
Relationship between precipitation and T on rainy days. P: precipitation, T: air temperature.

Rainfalls occurred on 41 days in the rainy season during the study period. Figure 5 shows the fitting relationships between ∆*W* and precipitation under different treatments on rainy days. The R^2^ of the fitting curves indicates that the precipitation explained 56.4%, 45.2%, and 58.0% of the variation in rainy days’ ∆*W* in NC, CR, and OR, respectively. When ∆*W* was equal to zero, the precipitation thresholds were 2.09, 2.54, and 2.22 mm at the daily scale, respectively. As shown in Figure 6, precipitation was negatively correlated with air temperature (T) on rainy days, so lower precipitation often correlated with higher T, which caused more evapotranspiration (ET) loss of soil water. As a result, the large soil water loss that occurred due to high T when precipitation was lower than the threshold provides further explanation for the fact that ∆*W* < 0 in these cases.

**Figure 7:**
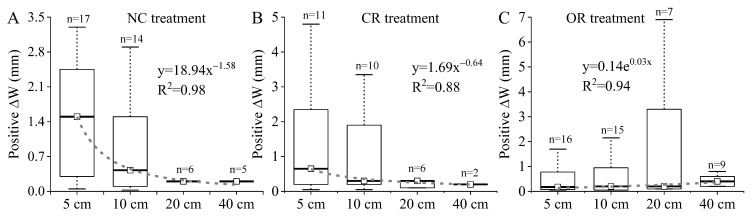
The positive ∆*W* on rainy days at different depths in different treatments. (**A**–**C**) represent the NC, CR, and OR treatments, respectively. A positive ∆*W* means an increase in *W* induced by precipitation on rainy days. NC: natural condition, CR: canopy + roots, OR: only roots. The gray dashed lines are the fitting curves for the median value of positive ∆*W* at different depths.

In order to evaluate the replenishment of the soil water via precipitation, the positive values of ∆*W* on rainy days were used to express the increase in W’s response to precipitation. Figure 7 shows the amplitude of positive ∆*W* at different soil depths with different treatments. In the NC and CR treatments, the median value of the amplitude of positive ∆*W* had a power function relationship with the soil depth, which decreased sharply in NC treatment from 1.5 mm at 5 cm depth to 0.43, 0.20, and 0.20 mm at 10, 20, and 40 cm depth, respectively, and it decreased slightly in the CR treatment from 0.65 to 0.30, 0.30, and 0.20 mm, respectively. The number of days of positive ∆*W* responses to precipitation also decreased rapidly with an increase in soil depth, only 6 days at 20 cm depth and 5 and 2 days at 40 cm depth in NC and CR, respectively. This indicates that precipitation did not effectively recharge soil water below 20 cm in the NC and CR treatments. However, the median value of the OR treatment exponentially increased from 0.18 mm at 5 cm depth to 0.2, 0.2, and 0.4 mm at 10, 20, and 40 cm depth, respectively. The response days were 7 and 9 at 20 and 40 cm depth, respectively.

### 3.5. The W and ∆W at the Monthly Scale

**Figure 8:**
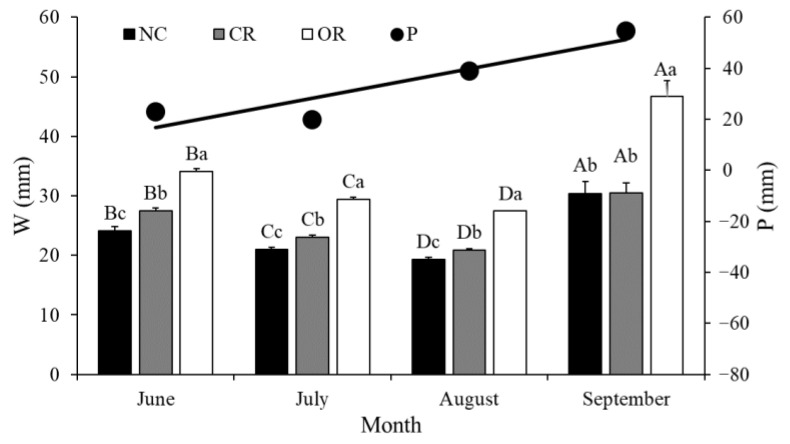
Comparisons of *W* and precipitation at the monthly scale. Different capital letters show a significant difference at *p* < 0.05 between different months under the same treatment, and different small letters show significance between different treatments in the same month. P: precipitation, *W*: soil water storage, NC: natural condition, CR: canopy + roots, OR: only roots.

The differences in *W* between different treatments at the monthly scale and monthly precipitation are shown in Figure 8. Monthly *W* always showed the order of OR > CR > NC each month, with significant differences (*p* < 0.05) being found, except for an insignificant difference between NC and CR in September. This indicated that removing the canopy was useful to increase the soil water. For the same treatment, the monthly *W* had significant differences between each month (*p* < 0.05), with a trend of September > June > July > August. The largest loss of soil water occurred in August, and the most effective replenishment of soil water via precipitation depended on the late stage of growth season in September. Meanwhile, monthly precipitation exhibited its highest value of 54 mm in September, followed by August (38.8 mm) and June (22.8 mm), and July showed the lowest value of 19.8 mm.

**Figure 9:**
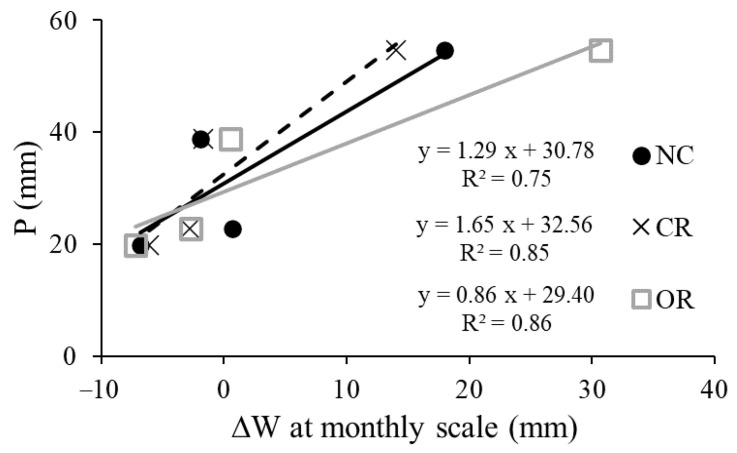
Responses of ∆*W* to precipitation under different treatments at the monthly scale. P: precipitation, ∆*W*: the change in soil water storage.

Figure 9 shows the response of ∆*W* to precipitation at the monthly scale. The monthly ∆*W* was positively correlated with precipitation under all three treatments. The explanatory power of monthly precipitation on the variation in monthly ∆*W* was 75.0%, 85.0%, and 86.0% in NC, CR, and OR, respectively, which were far higher than those at the daily scale. When the monthly ∆*W* was equal to zero, the thresholds of monthly precipitation were 30.78, 32.56, and 29.40 mm in NC, CR, and OR, respectively. The OR treatment showed the largest contribution of precipitation and the lowest precipitation threshold to increase the soil water at the monthly scale. In addition, high monthly precipitation amplified the differences in monthly ∆*W* between different treatments, indicating that the function of roots in soil water was strengthened by heavy precipitation during a month.

### 3.6. The Effect of Different Treatments on ∆W through the Whole Rainy Season

**Figure 10:**
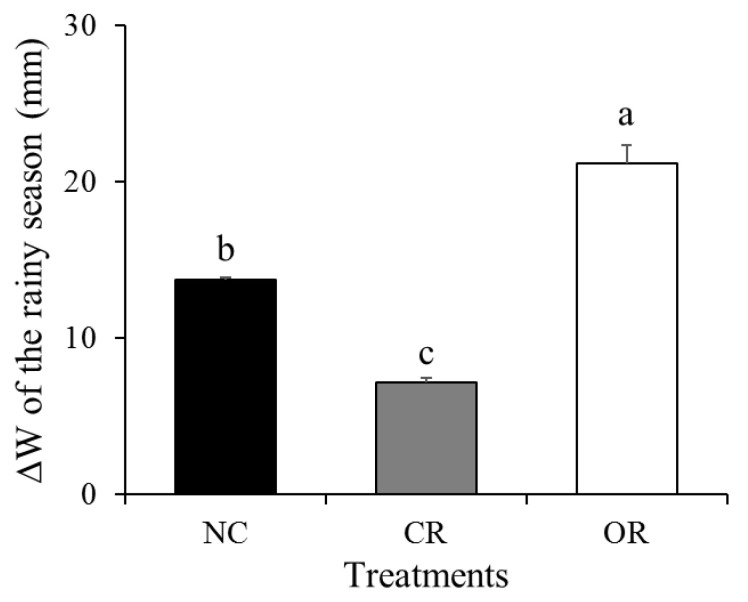
∆*W* of the whole rainy season under different treatments. Different small letters show significance (*p* < 0.05) between different treatments. ∆*W*: the change in soil water storage, NC: natural condition, CR: canopy + roots, OR: only roots.

The ∆*W* of the whole rainy season should be calculated by cumulative daily ∆*W*, namely the final day *W* minus the initial day *W*. To eliminate the influence of daily precipitation to soil water on a certain day as much as possible, the last 7 days in September and the first 7 days in June were selected, both of which had no daily precipitation or were below the threshold. As is shown in Figure 10, the ∆*W* of the whole rainy season showed a trend of OR > NC > CR, which had values of 21.16, 13.76, and 7.13 mm, respectively, and the differences reached a significant level (*p* < 0.05).

## 4. Discussion

The observation data of this study show that the total precipitation amount was 136 mm during June to September of 2015 (Figure 8). Based on the historical data from 1951–2016, this area had the lowest precipitation amount of approximately 231 mm from June to September (from Section 2.1). Thus, the study period occurred during an extreme drought year, which was very suitable for studying the response mechanisms of precipitation–soil and water–plants under drought stress.

### 4.1. Effects of Precipitation on Soil Water at Different Time Scales

Precipitation, as the main and unique source of soil water in the Loess Plateau, was responsible for the replenishment of soil water [23]. In this study, we found that the change in *SMC_m_* was positive and agreed with the daily precipitation, and the peaks of the *SMC_m_* always occurred after rainfall ended (Figure 2). Before September, with daily precipitation < 10 mm, the sensitivity of SMC to precipitation gradually decreased and the delay time gradually increased with increasing soil depths (Figure 3). This indicates that the effects of precipitation on SMC gradually decrease with soil depths, and a similar conclusion was found in grassland in the Loess Plateau [9]. Moreover, daily precipitation can only immediately affect the SMC at 0–10 cm depth that day, and precipitation greater than 10 mm might be the threshold to trigger the delayed response of the SMC at depths below 20 cm (Figure 3). It was found that 5–14 mm of precipitation was required to change the SMC at 10 cm depth for grassland and forestland in the Loess Plateau [10,12], and more than 55 mm of precipitation was needed below 50 cm depth in the typical steppe [14].

High precipitation causes larger differences in soil water between different treatments. For example, the OR treatment showed a different trend of SMC dynamic at the end of the observation period (Figure 2 and Figure 3D). This can be explained by the delayed infiltration process after the end of the high and continued precipitation of previous days. However, the delayed infiltration was not found in NC and CR treatments, because soil water was easily depleted by the transpiration process of the canopy [17,18]. Soil water was mainly controlled by the replenishment of precipitation and the loss of evapotranspiration [10,25,26], so the different trend in OR treatment also indicated that the evaporation was lower than the infiltration induced by previous precipitation at the end of the study period. Based on the results of Figure 3, we found that the evaporation and transpiration of soil water after rainfall was mainly from the 0–20 cm depth layer [27], while the infiltration below 20 cm could be retained after high and continued precipitation (>10 mm) without the transpiration process. The canopy in the NC and CR treatments impeded the effective infiltration of precipitation [16,17,25] and the roots had a promoting effect [28] for high precipitation. The larger differences in *W* and ∆*W* in September at the monthly scale (Figure 8 and Figure 9) also verify this.

Low precipitation might not effectively replenish soil water and may not even prevent a decrease in soil water. This study found that the response of soil water to precipitation exhibited a threshold phenomenon at different time scales. The lowest threshold values to increase soil water were 2.09–2.54 mm at daily scales (Figure 5) and 29.40–32.56 mm at the monthly scale (Figure 9). Another study also found that rainfall events <7.0 mm were insufficient to improve soil water [14]. This threshold phenomenon and the decline in soil water on rainy days could be explained by the loss of evapotranspiration, vegetation interception, runoff, and other hydrological processes [19,29]. Low precipitation is often coupled with high air temperature (Figure 6), which causes more evapotranspiration than on rainy days [30]. As a result, the rainfall infiltration cannot offset the water consumption of evapotranspiration in this case, leading to a decrease in soil water on rainy days.

The contributions of precipitation to soil water depend on time scales. We found that precipitation contributed more explanatory power to the ∆*W* variation at the monthly scale (75–86%) than at the daily scale (45–58%). This is because no-rain days reached 62 days, accounting for more than 60% of the total period, which might have daily ∆*W* > 0 mm due to the delayed effect of early precipitation or daily ∆*W* < 0 mm due to evapotranspiration losses. Coupled with the threshold phenomena of precipitation, the effect of precipitation on soil water was weakened and uncertain at the daily scale. However, in the long term, the delayed effect of rainfall infiltration was considered and was associated with the cumulative effect of precipitation on soil water, which caused the larger contribution at the monthly scale. In addition, the high and continued precipitation in September contributed the most to replenishing the soil water, and the low evapotranspiration induced by low air temperature and weak plant activity at the end of the growing season [25,26,31] also allowed for more retention of soil water.

### 4.2. Effects of Shrub Components on Soil Water Conservation

Plant components showed significant influences on soil water. The *SMC_m_* and monthly *W* both showed an order of OR > CR > NC (Figure 2 and Figure 8), indicating that the aboveground canopy failed to effectively increase the soil water and instead consumed more water under drought stress in extreme drought years. Based on another study in this area [25], the shrub canopy had a cumulative transpiration amount of 84–123 mm in a drier season, accounting for 56–80% of the total precipitation. It also had about 28–34 mm of canopy interception, leading to lower soil water under canopy cover. The underground roots had positive effects on soil water in soil water conservation [21,22], and the highest soil water being in the OR treatment verified this conclusion. Cui et al. [28] found that roots gradually play a more important role in the soil infiltration rate over infiltration time, which also provides support for the increasing trend of soil water in the OR treatment at the end of the study period (Figure 2). Wang et al. [22] compared the function of roots with rootless treatments in soil water and found that the roots decreased the propagation time of the wetting front by 33–113% and increased the amount of soil water in the saturation zone by 12–19%. The surface litter had different effects on soil water in this study. The lowest soil water in the NC treatment showed a negative effect, which might be explained by the fact that the litter could intercept a relatively lower rainfall and then increase the difficulty in soil water replenishment [11]. However, the litter showed a positive effect due to the higher ∆*W* of the rainy season in NC than in CR. This is because when daily P > 10 mm happened, the litter would transfer the negative interception of rainfall infiltration to the positive storage of rainfall and runoff, increasing the infiltration time of the soil water [20,32].

Plant components also had an important influence on the response of soil water to precipitation. In the OR treatment, precipitation showed the highest contribution to and relative impact on soil water, and the depths of precipitation recharging soil water more easily reached below 20 cm (Figure 7). Thus, the roots strengthened the response because the transport paths and channels provided by the roots effectively enhanced the convenience of the infiltration and ET processes [21,22,33]. In the CR treatment, precipitation showed the lowest contribution to and relative impact on soil water at the daily scale, and only on 2 days was the response of soil water to precipitation triggered. Thus, the canopy weakened the response due to the great disturbance to hydrological processes [17,18]. In the NC treatment, precipitation had different effects on soil water. The litter had a varied effect on the response relationship, where it strengthened the interception function under low precipitation based on the significant difference between NC and CR before September. However, the litter offset a part of the weakening function of the canopy under high precipitation based on the similar values between NC and CR in September (Figure 8). The litter played a neutral role in regulating the soil water response to precipitation, due to its function involving intercepting and accumulating rainfall and runoff, reducing evaporation, improving soil properties, increasing infiltration, etc. [11,20,32].

Consequently, soil water and its response to precipitation were strengthened by the roots, weakened by the canopy, and neutralized by the litter. Thus, regular cutting of the canopy at the single-plant scale may help to increase soil water by decreasing evapotranspiration and interception. Similar results can be achieved by reducing canopy of trees [34], but a 41% infiltration rate reduction was found when the vegetation in the grassland was cut [35]. However, at the stand scale, the effect of cutting plants on soil water depends on the thinning intensity and vegetation types [36,37]; for example, a 50% thinned shrub had higher soil water storage than a 100% thinned grass [36]. The conclusion in this paper was limited to the single-shrub scale. Further research is needed at a larger scale due to the complex hydrological processes involved. Nevertheless, this study on the function of shrub components in precipitation and soil water response relationships is still useful for vegetation management and hydrologic regulation.

## 5. Conclusions

The study period, June to September of 2015, was an extreme drought season. Soil water showed complex responses to precipitation under typical shrubs at different time scales in the Loess Plateau, and the shrub components played different roles.

(1) The response of the SMC to daily precipitation was gradually decreased and delayed for longer with an increase in soil depth. It was delayed by 0–2 days at a 10 cm depth, 5 days at a 20 cm depth, and 8 days or longer at a 40 cm depth.
(2) A threshold phenomenon existed in the response of soil water to precipitation. Daily precipitation > 10 mm was the threshold to trigger a delayed response of the SMC below a 20 cm depth. The thresholds of precipitation to increase *W* were 2.09–2.54 mm at the daily scale and 29.40–32.56 mm at the monthly scale, respectively.
(3) The contribution of precipitation to the soil water was 75–86% at the monthly scale, which was much larger than at the daily scale (45–58%).
(4) Plant components showed significantly different influences on soil water. The roots could improve the soil water contents and strengthen its response to precipitation. The canopy consumed soil water, causing the smallest ∆*W* of the rainy season to be in the CR treatment and greatly weakening the influence of precipitation on soil water. The litter played a neutral role in the response of *W* to precipitation, decreasing *W* when daily precipitation was <10 mm and increasing *W* when daily precipitation was >10 mm.

## Figures and Tables

**Table 1:** The correlation between *W* and ∆*W* of every day with precipitation during the rainy season.

Indicators	Soil Depths (cm)	NC	CR	OR
*W*	5	0.161	−0.018	−0.028
	10	−0.047	−0.046	−0.054
	20	−0.255 **	−0.226 *	−0.182
	40	−0.216 *	−0.182	−0.223 *
	Total (0–40)	−0.043	−0.125	−0.167
∆*W*	5	0.477 **	0.301 **	0.311 **
	10	0.403 **	0.298 **	0.391 **
	20	−0.162	−0.054	−0.048
	40	0.239 *	0.238 **	0.325 **
	Total (0–40)	0.504 **	0.419 **	0.384 **

* and ** indicate significance at the *p* < 0.05 and *p* < 0.01 level, respectively. *W*: soil water storage, ∆*W*: the change in soil water storage, NC: natural condition, CR: canopy + roots, OR: only roots.

## Data Availability

All data from this study have been included in the text.
